# Adjuvant chemo-radiotherapy in the “sandwich” method for high risk endometrial cancer—a review of literature

**DOI:** 10.1186/s12905-015-0207-0

**Published:** 2015-06-24

**Authors:** Yachun Bie, Zhenyu Zhang, Xiaolan Wang

**Affiliations:** Department of Obstetrics and Gynecology, Beijng Chao-yang Hospital affiliated to Capital Medical University, No. 8 Gongti South Road, Beijing, 100020 P. R. China; Department of Obstetrics and Gynecology, China Meitan General hospital, Beijing, P. R. China

**Keywords:** Endometrial cancer (EC), Sequential chemotherapy and radiotherapy, Disease free survival (DFS), Overall survival (OS)

## Abstract

**Background:**

Endometrial cancer is a common female malignancy. Patients with high-risk endometrial cancer have relatively high incidence of metastasis and recurrence. Despite complete resection, patients with stage III or IV are at high risk of local or distant recurrence. Systemic adjuvant treatment includes chemotherapy and radiotherapy. But the optimal scheduling is not known. Recently proposed sequential chemo-radiotherapy as sandwich therapy for high risk endometrial cancer have yielded encouraging results. This article is to review the adjuvant chemo-radiotherapy in the “sandwich” method for high risk endometrial cancer to help clinicians identify the most effective adjuvant treatment for patients with high risks of it.

**Methods:**

We used MEDLINE, EMBASE, Cochrane Library and CBM databases to search the literature.

**Results:**

A systematic review was made. And most data showed “sandwich” therapy is feasible, efficacious, well-tolerated and resulted in excellent long-term progression free and overall survival in the setting of advanced endometrial cancer.

**Conclusion:**

Randomized trials are necessary to compare chemo-radio therapy given in the “sandwich” fashion to other means of sequencing these treatment modalities. It is also necessary to define which population is best suited for “sandwich” adjuvant therapy.

## Background

Endometrial cancer is a common female malignancy. The incidence varies because of different lifestyles and regions. In developed countries, the incidence rate is the highest among female genital malignancies, and the age of patients become younger. The prognosis is closely related to the disease stage. If the diagnosis is during stage I, then the survival rate is about 90 %. But those with extra-uterine disease (stage III or IV) have a significant risk of death despite current therapies, with 5- year survival rates ranging from 23 to 72 % [1–4].

Despite complete resection, patients with stage III or IV are at high risk of local or distant recurrence. Systemic adjuvant treatment includes chemotherapy and radiotherapy. But the optimal scheduling is not known. Recently proposed sequential chemo-radiotherapy as sandwich therapy for high risk endometrial cancer have yielded encouraging results, while a single center experience shows that “sandwich chemo-radiotherapy” seems to be more toxic particularly for patients who had pelvic and para- aortic irradiation. Therefore, it might be more convenient to delay radiotherapy after six cycles of chemotherapy for patients with the indication of pelvic para-aortic radiotherapy [5].

The evaluation of high risks are according to patients’ age, pathology grade, stage, histology type, lymphovascular space invasion (LVSI), tumor size, myometrial invasion, parametrial involvement, cervical stroma or vaginal disease and positive pelvic or para-aortic nodes (Table 1). Patients with high-risk endometrial cancer have relatively high incidence of metastasis and recurrence. Therefore, adjuvant chemotherapy and radiotherapy after surgery is essential to reduce the risk of relapse.

**Table 1:** Characteristics of patients with high risk endometrial cancer

Characteristics of patients	High risk endometrial cancer	Low risk endometrial cancer
Age	≥60	<60
Grade	≥2	<2
Stage	III-IV	I-II
Histology type	serous and clear cell carcinoma	endometrioid carcinoma
Lymphovascular space invasion (LVSI)	Yes	No
Tumor size	≥1/2 uterine cavity	<1/2 uterine cavity
Myometrial invasion	≥1/2	<1/2
Parametrial involvement	Yes	No
Cervical stroma or vaginal disease	Yes	No
Positive pelvic or paraaortic nodes	Yes	No

High-risk endometrial cancer criteria included, but were not limited to these

Adjuvant chemo-radiotherapy in the “sandwich” method for high risk endometrial cancer has been described consisting of initial chemotherapy of limited duration, followed by radiotherapy, and then subsequent consolidation chemotherapy again (CRC).

We made a systematic review about the adjuvant chemo-radiotherapy in the “sandwich” method for high risk endometrial cancer after surgery to help clinicians in identifying the most effective adjuvant treatment for patients with high risks.

## Methods

### Search strategy and selection criteria

**Fig. 1:**
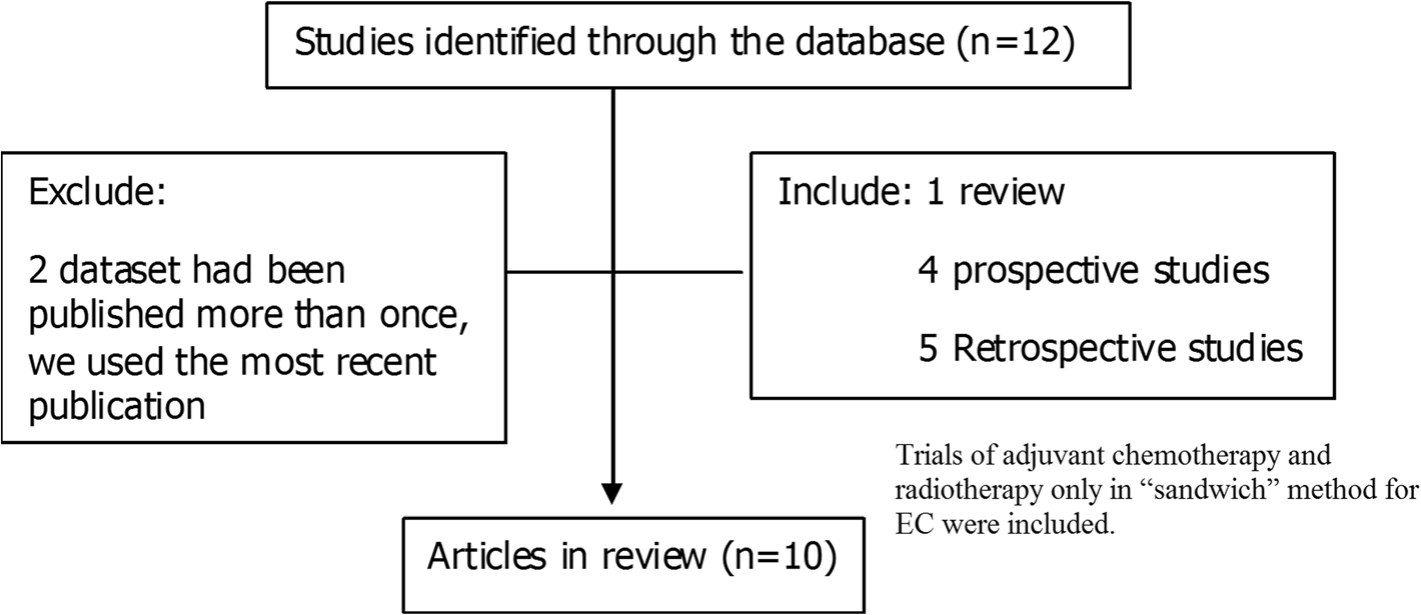
Selection process for articles included in the systematic review

We systematically searched MEDLINE, EMBASE and Cochrane Library databases (from their commencements to February 2014), with no language restriction, for studies in women of the association between endometrial cancer and adjuvant chemotherapy and radiotherapy in the “sandwich” method. Index words included the medical subject headings (MeSH) endometrial neoplasms and uterine neoplasms, and the following text words: endometrium, endometrial, uterus, uterine, cancer carcinoma, chemotherapy, radiotherapy and adjuvant therapy. Trials of adjuvant chemotherapy and radiotherapy only in “sandwich” method for EC were included. Search terms related to study design and publication type included systematic review, clinical trial, meta-analysis, controlled clinical trials, and randomized controlled trials. Reference lists of identified studies were scanned for additional citations until no additional articles could be identified. Subjects underwent surgical staging comprised of total hysterectomy, bilateral salpingo-oophorectomy, bilateral pelvic and para-aortic lymph node dissection and peritoneal cytology. If a dataset had been published more than once, we used the most recent publication (Fig. 1).

## Results

There were 10 articles included in this review. Four of them are prospective and five are retrospective studies (Table 2). Another one is a review.

**Table 2:** Summary of study details and patient characteristics from recent “sandwich” regimens

Study	Design	Drugs	N	Age (median and range)	Stage	Subtype (%)			
						EC	SC	CC	Mixed
Lupe [6]	P	paclitaxel/carboplatin	43		IIIA-IVB	37	35	7	6
Geller [7]	P	docetaxel/carboplatin	41	59	IIIA-IVB	78	10		2
Einstein [8]	P	ifosfamide/cisplatin	27		I-IV uterine carcinosarcoma		100		
Fields [9]	P	paclitaxel/platinum	30	69(45–82)	I-IV uterine papillary serous carcinoma (UPSC)		100		
Secord [10]	R	paclitaxel/carboplatin 79 %	45	62(35–83	III-IV	48	17	5	28
Geller [11]	R	taxane/carboplatin	23	57(28–78)	IIA-IVB(78 %III,13 %IV)	43	52	4	
Abaid [12]	R	paclitaxel/carboplatin	32	67	IA-IVA	59	13	9	9
Lan [13]	R	docetaxel/carboplatin	35	53(23–69)	IIIA-IVB	83			
Dogan [5]	R	paclitaxel/carboplatin	11	63(36–83)	IIIC	73	18	9	

*P* Prospective Study, *R* Retrospective Study, *N* number, *EC* endometrioid adenocarcinoma, *SS* serous carcinoma, *CC* clear cell carcinoma

### Prospective studies

Lupe *et al.* did a prospective cohort study to determine the feasibility of adjuvant paclitaxel and carboplatin chemotherapy with involved field radiotherapy for 43 patients with stage III or IV uterine malignancies (40 carcinomas and 3 mixed Mullerian tumors, MMT’s). They concluded that therapy in this way is associated with a low rate of local recurrence and favorable survival for advanced endometrial cancer. They previously reported the toxicity of this protocol. About 31 % of their patients experienced grade 3 or 4 toxicity with peripheral neuropathy and neutropenia [6].

Geller *et al.* did a phase II trial of carboplatin and docetaxel followed by radiotherapy given in “Sandwich” way for advanced and recurrent patients. They believed that docetaxel instead of paclitaxel decreased neurotoxicity. And patients had minimal delay between treatment modalities [7].

The low rate of recurrence in the radiation field was notable in both studies.

Another study did by Einstein *et al.* about phase II trial of adjuvant pelvic radiation “sandwiched” between ifosfamide or ifosfamide plus cisplatin in women with uterine carcinosarcoma [8]. The 2 year DFS was similar in both groups. The addition of cisplatin added toxicity without improving efficacy. With ifosfamide alone, the efficacy of the “sandwich” therapy comes with a moderate but tolerable toxicity profile.

Fields *et al.* also had a prospective study on patients with uterine papillary serous carcinoma (UPSC) to treat with pelvic radiation “sandwiched” between six cycles of paclitaxel(T)/platinum(P) chemotherapy. They also showed the radiation “sandwiched” between T/P chemotherapy is a well-tolerated and efficacious regimen for patients with completely resected UPSC [9].

### Retrospective studies

There are also 5 retrospective studies to assess the sequential chemotherapy and radiotherapy as “sandwich” therapy for the treatment of high risk endometrial cancer.

A multicenter retrospective analysis was done by Secord. Similar to other studies, it showed that the most common grades 3–4 chemotherapy toxicities were neutropenia (33 %), anemia (19 %), and neurotoxicity (33 %) in women treated in the “sandwich” manner. They proved that sequential CRC (chemotherapy-radiotherapy-chemotherapy) was associated with improved survival in women with advanced stage disease compared to other sequencing modalities with a similar adverse effect profile [10].

Geller, *et al.* also conducted a retrospective analysis. There were 23 patients of advanced stage of EC, the majorities were in stage III(78 %) and histological types were serous (52 %), treated with comprehensive surgical staging followed by adjuvant therapy in “sandwich” method. It consisted of sequential chemotherapy and pelvic radiation with or without para-aortic radiation. It was feasible, well tolerated and resulted in excellent long-term progression free and overall survival [11].

Abaid, *et al.* analyzed 32 endometrial cancer patients who were treated with carboplatin and paclitaxel. 186 cycles had been administered and 94 % of patients completed the planned. The incidence of grade 3 neutropenia is 3.1 % and no incidence of grade 4 neutropenia. Grade 3 anemia in 12.5 % of patients and grade 4 in 3.1 % of patients were observed. They proved the hematologic toxicity was well tolerated and non-hematologic toxicity was mild and easily managed. [12].

Lan, *et al.* retrospectively reviewed patients with staged III - IV disease who received adjuvant chemotherapy (docetaxel plus carboplatin) administered alone or interposed with radiotherapy. In all 35 patients, 25 patients with stage IIIC - IVB disease and 1 patient with stage IIIA disease received radiotherapy sandwiched between chemotherapy cycles (total, three to six cycles). They showed the sandwich therapy is efficacious and well tolerated for stage IIIC - IVB endometrial cancer. And they also showed adjuvant chemotherapy alone with docetaxel and carboplatin might be sufficient for stage IIIA disease [13].

Most data supports that sandwich therapy is feasible, efficacious, well tolerated and resulted in excellent long-term progression free and overall survival in the setting of advanced endometrial cancer.

While Dagon, *et al.* got an opposite conclusion. They compared “sandwich therapy” with six cycles of chemotherapy followed by adjuvant radiotherapy with respect to tolerability and acute toxicity. 25 patients with stage IIIC endometrial cancer were treated with either three cycles of paclitaxel (175 mg/m2) and carboplatin (AUC 6) on a q21-day schedule followed by irradiation (45–50.4 Gy) or six cycles of the same chemotherapy followed by radiotherapy. 11 patients had sandwich therapy and 14 patients were treated by 6 cycles of chemotherapy followed by radiotherapy. Three out of the five patients who could not complete in the sandwich therapy group had pelvic and para-aortic radiotherapy. In sandwich group, acute radiotherapy related grade 1–2 gastrointestinal and genitourinary system toxicities were observed in 72.8 and 63.6 % of patients, respectively. Undesired treatment breaks in the course of radiotherapy were also observed in six patients for this group and all of them had pelvic and para-aortic radiotherapy. They concluded the sandwich chemo-radiotherapy seems to be more toxic particularly for patients who had pelvic and para-aortic irradiation. The author suggests that it is a good choice to delay radiotherapy after all cycles of chemotherapy for patients with para-aortic radiotherapy [5].

Dagon, *et al.* also showed the median follow-up was 18 months (range 12–53 months). A patient had sandwich therapy was detected of vaginal vault recurrence at 17 months after primary treatment [5].

In these “sandwich” regimens, every patient accepted pelvic radiotherapy (Table 3). Fields were extended and addition of HDR (high doses radiotherapy) vaginal vault brachytherapy was left to the discretion of the treating radiation oncologist.

**Table 3:** Summary of radiation details in “sandwich” regimens

Study	N	Pelvic radiotherapy (RT)	Method	Totle cycles of CT
Lupe [6]	43	45Gy	4CT+RT+2CT	6CT
Geller [7]	41	45Gy	3CT+RT+3CT	6CT
Einstein [8]	27	45Gy	3CT+RT+3CT	6CT
Fields [9]	30	45Gy	3CT+RT+3CT	6CT
Secord [10]	45		3CT+RT+CT	6 ~ 9CT
Geller [11]	23	45Gy	2/3/4CT+RT+CT	2 ~ 4CT
Abaid [12]	32	40–46Gy	3CT+RT+3CT	6CT
Lan [13]	35	44–64Gy	1/2/3/4/5CT+RT+CT	3 ~ 6CT
Dogan [5]	11	45–50.4Gy	3CT+RT+3CT	6CT

*RT* radiotherapy, *CT* chemotherapy; Radiation therapy dosage and irradiated fields were determined by disease site, lymph node status and the discretion of the treating radiation oncologist

Tables 4 and 5 show summary of study details with associated outcomes from recent “sandwich” regimens.

**Table 4:** Summary of outcomes from recent “sandwich” regimens

Study	N	Completed (%)	Recurrence (%)	Neutropenia (III-IV) (%)	Neuropathy (%)	3 year DFS/PFS (%)	3 year OS (%)
Lupe [6]	43	81	49			53	68
Geller [7]	41		24	19	5	71	90
Einstein [8]	27	70	37	18	1		
Fields [9]	30	97	38	42		54	52
Secord [10]	45		24	33	33	69	88
Geller [11]	23					80	88
Abaid [12]	32	94	16	3	3	84	
Lan [13]	35	74	35		8.6	73	87
Dogan [5]	11	55	9	64	82		

*PFS* Progression Free Survival, *DFS* Disease Free Survival, *OS* Overall Survival

**Table 5:** Summary of recurrence from recent “sandwich” regimens

Study	N	Recurrence (%)	Local recurrence (%)	Distant recurrence (%)
Lupe [6]	43	49	5	44
Geller [7]	41	24	2	22
Einstein [8]	27	37	15	22
Fields [9]	30	38	7	31
Secord [10]	45	24	4	20
Geller [11]	23			
Abaid [12]	32	16	9	7
Lan [13]	35	35	8	27
Dogan [5]	11	9	9	0

From Table 5, the low rate of local recurrence is notable after the sandwich therapy for high-risk endometrial cancer patients.

## Discussion

For high-risk endometrial cancer, we have not yet achieved optimal outcomes with currently available therapies [14]. Using chemotherapy alone has been associated with high pelvic relapse rates. The use of adjuvant chemo-radiotherapy seems to be promising with acceptable recurrence rates.

In theory, sequential of both radiation and chemotherapy modalities should limit the overall toxicity and allow for maximum therapeutic dosing. However, there is no consensus regarding modality of adjuvant chemotherapy and radiation in women of high risk EC. The most studies showed the sandwich approach to treating high risk EC patients is feasible, even patients with advanced age and late stage.

In past studies, most patients were treated with paclitaxel and carboplatin chemotherapy. Many physicians have already adopted the carboplatin and paclitaxel as standard regimens. Other regimens included ifosfamide and cisplatin.

In our review the low pelvic relapse rate associated with “sandwich” treatment protocol was found. But comparing survival rates is a problem in light of the substantial differences between patient populations selected for each study. The different conclusions of tolerability, acute toxicity, recurrence, DFS and OS rates in sandwich therapy in high risk of EC may be due to the different histological subtype, dose of therapy and the combination ways of radiotherapy.

A larger multi-institutional clinical trial should be considered to confirm these pilot data.

## Conclusion

The “sandwich” therapy for high risk EC appears to be tolerable and effective. Future randomized trials are necessary to compare chemo radiotherapy given in the “sandwich” fashion to other means of sequencing these treatment modalities. For example the randomized trial can be designed to compare “sandwich chemo-radiotherapy” with all cycles of chemotherapy followed by adjuvant radiotherapy with respect to tolerability and acute toxicity. And it is also necessary to define which population is best suited for “sandwich” adjuvant therapy.
